# Diagnosing and Managing Linear Scleroderma in a Low-Resource Setting

**DOI:** 10.1155/2023/3918638

**Published:** 2023-08-17

**Authors:** Sreyleak Luch, Pauravy Men, Gwenyth Fischer, Andrew Wu

**Affiliations:** ^1^Chenla Children's Healthcare, Sangkat Krachech, Krong Krachech, Kratie Province, Cambodia; ^2^Department of Pediatrics, Pediatric Critical Care Medicine, University of Minnesota, Minneapolis, MN, USA; ^3^Division of Critical Care Medicine, Department of Anesthesiology, Critical Care and Pain Medicine, Boston Children's Hospital, Boston, MA, USA

## Abstract

**Background:**

Linear scleroderma is one of the five forms of scleroderma, but it is the most common form of localized scleroderma in childhood. If left untreated, it can lead to severe disfigurement and functional impairment. The typical appearance is a linear streak with cutaneous induration on the face or head in association with various ophthalmological and neurological signs and symptoms. Treatment typically includes corticosteroids and/or methotrexate with life-long monitoring for recurrence. *Case Presentation*. A 12-year-old girl presented to our clinic in northern rural Cambodia with a history of a linear streak on her forehead that was growing down her nasal bridge. She denied any tenderness or family history of rheumatic disease. Her history was significant for strabismus as a child. A visiting pediatric rheumatologist assisted us with the appropriate diagnosis and treatment plan.

**Conclusion:**

In our case report, we present a child with linear scleroderma who fortunately came to medical attention early and received appropriate treatment before the onset of complications. She was treated with systemic immunosuppression as well as topical steroids. After treatment, she had no further progression on her face and continued to follow up with us to monitor for disease activity. To summarize, linear scleroderma is an uncommon diagnosis for general pediatricians and should be recognized early to provide appropriate treatment and follow-up.

## 1. Introduction

Scleroderma is a rare (3 cases per 100,000 individuals annually), autoimmune disease that causes sclerotic changes in the skin [1]. It is usually self-limiting but can leave cosmetically disfiguring and functionally impairing consequences [1, 2].

Linear scleroderma (“linear morphea” or “en coup de sabre”) is one of the five forms of scleroderma and the most common type of localized scleroderma in childhood [1]. It was first described by Addison et al. in 1854, where females demonstrated a higher incidence than males (ratio F : M 4 : 1) [3]. The diagnosis of localized scleroderma is based on clinical findings, specifically by the appearance of the lesion, which typically involves progressive loss of subcutaneous fat with pigmentation changes that appear as shiny, thick patches of the skin. It usually presents with at least one linear streak of cutaneous induration that may only involve dermis but may also extend to underlying structures such as subcutaneous tissue, muscle, and bone leading to cosmetic and functional disability. It can affect one side of the face or head in the frontoparietal region. The age of presentation in children is usually six to eight years of age on average but can range from birth to 17 years. Associated symptoms may include joint contracture and various ophthalmological and neurological abnormalities. The pathogenesis remains unknown, though some proposed risk factors include coexisting autoimmune processes, trauma, insect bite, psychological stress, and vascular abnormalities [4, 5]. The differential diagnosis for linear scleroderma includes localized lipodystrophy, morphea-like lesions of phenylketonuria, graft-versus-host disease, eosinophilic fasciitis, erythema migrans, eosinophilic fasciitis, port-wine stains, and connective tissue nevi [5]. Linear scleroderma can often be confused with Parry–Romberg syndrome, which also involves loss of subcutaneous fat in a facial region in children [6]. However, Parry–Romberg syndrome typically involves the optic nerve and atrophy of the bone and muscles, which linear scleroderma does not. Given the slow and benign nature early on in the course of linear scleroderma, treatment can foreseeably be delayed especially among children living in rural or otherwise low-resource settings where medical care is sparse. In addition, reports on linear scleroderma in children are rare as many reports are in older patients or adults [7]. The objective of this case report is to present a pediatric patient with a seemingly benign dermatological problem that was treated early before serious complications arose.

## 2. Case Presentation

A 12-year-old girl presented with her father to an outpatient clinic in northern Cambodia with a two-year history of a white rash extending down her forehead to her nose. The rash exhibited pitting and had been growing downwards along her nasal bridge. She had been seen by other local, rural medical providers but presented to us given her condition was not improving over the course of the past year. She denied any associated tenderness or pain and denied history of trauma in the associated area. There was no family history of rheumatic disease. She denied any functional limitations that seemed to be associated with the rash such as facial asymmetry, pain, or tightness when using facial expressions, vision changes, or headaches. She denied any stiffness, swelling, or pain in her hands, wrists, feet, or ankles.

Her medical history was notable for bilateral exotropia, for which she received treatment at 2 years of age with spectacles followed by surgery. She denied photosensitivity or vision changes.

**Figure 1:**
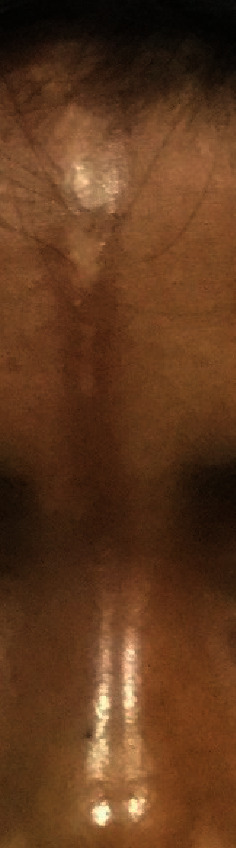
On presentation, the child exhibited a white shiny plaque with skin atrophy and alopecia extending from her frontal scalp to her nose. There was no evidence of ulceration, excoriation, redness, or tenderness.

Facial examination revealed a shiny, depressed, white, linear, nonpruritic plaque on the frontal aspect of her scalp extending to the nasal tip with associated skin atrophy and alopecia in the region of the plaque (Figure 1). There was no involvement of the oral mucosa though the gums, lips, and tongue. Other surrounding facial and bony structures were intact.

Lab tests were obtained and included a complete blood count with differential, erythrocyte sedimentation rate, urea, creatinine, alanine transaminase, rheumatoid factor, and lupus antibody latex test, which were all negative. Skin biopsy and ANA testing were not done due to laboratory test limitations. A chest radiograph and an abdominal ultrasound were normal. At the time, a formal consent process was not available but explicit verbal consent for publication and use of the photos was obtained.

Under the guidance of a visiting pediatric rheumatologist, the patient was clinically diagnosed with linear scleroderma.

Treatment began with prednisolone 1 milligram per kilogram per day for 30 days in addition to 1% hydrocortisone cream applied to relieve itchiness and xerosis. After one month, methotrexate was started (1 milligram per kilogram per week) and a steroid wean was initiated.

**Figure 2:**
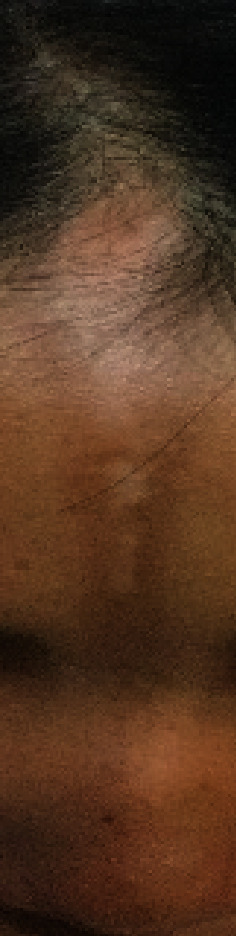
Two years later after treatment with corticosteroids and methotrexate. On this follow-up, the lesion is less shiny, though scarring and alopecia persisted. The lesion is less apparent on the nose.

The patient followed up in our clinic every two months while on methotrexate to monitor for progress. The skin lesion did not show signs of spread or skin depression. Scarring and alopecia persisted, but there were no signs of inflammation (Figure 2). Methotrexate was stopped after two years of treatment, and she continues to be followed up every six months to reassess the lesion for recurrence or flare ups.

## 3. Discussion

In this report, we present a child with linear scleroderma who was promptly diagnosed and treated. According to consensus recommendations by Zulian et al. in 2019, the diagnosis of linear scleroderma is typically done by evaluating clinical findings, followed by imaging tests to determine the extent of disease, all under the care of a pediatric rheumatologist [5]. Additional diagnostic tests may include skin biopsy if confirmation is needed, which may show mild acanthosis and collagen bundles replacing the adipose tissue [8]. Biological markers also contribute to the diagnostic probability. Anti-dsDNA, anticentromere, and antitopoisomerase antibodies are rarely positive [7]. In contrast, a high proportion of patients with localized scleroderma are antinuclear antibody (ANA) positive. In a report of 57 patients, 75% were ANA positive [7].

As linear scleroderma is an autoimmune phenomenon, children with this finding have a higher risk of developing other autoimmune disorders. For example, eosinophilia is sometimes seen in localized scleroderma, which led our team to entertain eosinophilic fasciitis (Shulman syndrome) as a possibility [9]. Fortunately, the eosinophil count in this case was normal. Extracutaneous involvement can occur in up to 20% of patients with linear scleroderma [5, 10], with articular involvement being the common. In our case, she had no signs of arthralgia or arthritis.

The involvement of our patient's face and head with the lesion is of some significance since CNS signs and symptoms may also be present in this subgroup of scleroderma. These signs and symptoms may include ocular changes, seizures, headache, behavioral changes, and learning disabilities. The most frequent ocular manifestation is uveitis, though reports have been published on a link between acquired strabismus and linear scleroderma as well [11, 12]. The child in this case had bilateral strabismus, which was recalled by family members as happening before the onset of this skin lesion, though timing between the lesion and ocular involvement has not been reported in the literature to our knowledge. Because of these potential associated symptoms and complications, evaluation by orthodontic, maxillofacial, and ophthalmological specialists is recommended as well [5]. In our low-resource setting, these specialists were unavailable. MRI is also highly recommended in patients with involvement of the head or face [5]. Unfortunately, performing an MRI in our setting would require ground transport over the course of several hours and was not practically feasible for both our low-resource hospital and this family.

The management of linear scleroderma may include the use of topical medications (corticosteroids and calcineurin inhibitors), immunosuppressive pharmacological agents (corticosteroids and methotrexate), physical therapy, and ultraviolet (UV) phototherapy [3, 12]. However, the most reported experience is with either oral or intravenous steroids in combination with methotrexate. Indeed, methotrexate is often the first line therapy and drug of choice in moderate to severe disease, with a dose of 1 mg/kg/week or 15 mg/m^2^/week [7, 10]. Long-term methotrexate (MTX) therapy is beneficial and well tolerated for localized scleroderma, as is steroids during the active phase of the disease when used as “bridge” therapy for the MTX [13]. Side-effects of MTX may include nausea, headache, and transient transaminitis [5]. In cases of resistance to methotrexate, mycophenolate mofetil has been used. UV therapy has been rarely done in children and was not feasible at our facility.

Linear scleroderma has a slow progression with resultant of atrophy and deformity before it becomes dormant within 2–20 years [1, 2]. However, it may recur after several years of quiescent disease, hence why our patient continues close follow-up. Follow-up is done every two to four months during the first one to two years after diagnosis. When the disease is under control, follow-up should be done every six months during the second year after the diagnosis and annually thereafter. In our patient, clinical improvement was demonstrated after two years of treatment as she demonstrated no further atrophy or spread of the lesion.

Given the very low-resource setting of our practice, we did not have imaging capabilities or the ability to detect auto-antibodies. However, we were fortunate to have a visiting pediatric rheumatologist present and consulting. We realize that not all low-resource settings will have this luxury; therefore, we hope that our report highlights both this disease well as the need for continued education in various specialized fields of medicine for those practicing medicine in low-resource settings.

Our report highlights a very rare general pediatric finding but a more classic rheumatologic one. Linear scleroderma can cause severe structural deformities if left untreated. Despite not having a wide variety of consultants and advanced laboratory resources available, we were still able to provide a proper diagnosis and treatment course. We hope to share awareness of this disease with other general pediatricians around the world who also practice in a similar setting, so they may also appropriately manage these patients before complications occur.

## Data Availability

The data used to support the findings of this study are included within the article.
